# United States military working dogs from 2019 to 2021: analysis of causes of service discharge and decreased service life

**DOI:** 10.3389/fvets.2025.1580628

**Published:** 2025-10-09

**Authors:** Dakota Discepolo, Brian Farr, Desiree Broach, Andrea Henderson, Angelina Gerardo, Jessie Dyer, Eric Best

**Affiliations:** ^1^U.S. Army Combat Capabilities Development Command Chemical Biological Center, Edgewood, MD, United States; ^2^U.S. Department of Energy, Oak Ridge Institute for Science and Education, Oak Ridge, TN, United States; ^3^Department of Defense Military Working Dog Veterinary Service, San Antonio, TX, United States; ^4^Department of Emergency Management and Homeland Security, University at Albany, Albany, NY, United States

**Keywords:** military working dog, service discharge, working dog, retirement, service life

## Abstract

**Introduction:**

Military working dogs (MWDs) are maintained by the United States Department of Defense (DoD) in effort to maintain readiness. MWDs provide valuable abilities which include explosive and drug detection capabilities as well as security support. However, acquiring, training, and maintaining MWDs requires significant investment of resources. Therefore, understanding the prominent causes of service discharge and associated causes and demographics associated with decreased service duration in the modern MWD population is crucial.

**Methods:**

To meet this objective, an extensive review was conducted of service discharge records of MWDs who were discharged from service in fiscal years 2019 through 2021. The causes of service discharge were categorized and subcategorized by Army Veterinary Corps Officers with extensive MWD experience. Service life and operational service life were calculated using lifecycle dates. Chi-square analysis compared frequencies of categories and subcategories, and logistic regression analysis was conducted on occurrence of the five most prominent categories to identify associations with breed, size, subpopulation, goal at procurement, outcome of service discharge, and duration of service.

**Results:**

The presented results include data on 1,230 MWDs who were discharged from service during the selection period. The five most prominent causes of service discharge were neuromusculoskeletal disease, training, fear-anxiety, neoplasia, and heat injury which accounted for discharge of 83.50% of the MWDs. Each of these prominent categories were significantly associated with at least one of the population characteristics analyzed and all of them were significantly associated with duration of service. ANOVA analysis comparing mean service life resulted in significant differences of mean overall service with main effects of breed (*p* = 0.0252), outcome (*p* = 0.0004), service discharge category (*p* < 0.0001), and subpopulation (*p* < 0.0001).

**Conclusion:**

These findings can inform mitigation strategies to prevent early or preventable service discharge in the future.

## 1 Introduction

Military working dogs (MWDs) are maintained by the United States Department of Defense (DoD) as a mechanism to provide explosive and drug detection as well as security support (1, 2). MWDs are acquired from worldwide breeders or from the DoD MWD Breeding Program (3). The Air Force’s 341st Training Squadron (341 TRS) is the entity responsible for procuring, training, and distributing MWDs to other services within the DoD (2). To acquire MWD trainees, teams of experienced personnel periodically travel to assess and select dogs who meet the selection criteria. Dogs are assessed for procurement between 1 and 3 years of age. The selection criteria include radiographic screening for congenital orthopedic issues, general health screening, temperament, environmental assessments, and aptitude assessments for substance search and human apprehension. Alternatively, the DoD MWD Breeding Program provides about 13% of the MWD population with approximately 50 to 90 Belgian Malinois and Dutch Shepherd puppies whelped each year (1). MWD candidates from the Breeding Program are raised in foster homes from 6 weeks to 7 months of age with periodic medical care, evaluation, and training sessions. These candidates return to the 341 TRS at 7 months of age for further evaluation and training prior to consideration for procurement at 12 months of age.

Upon arrival to the 341 TRS, the trainees not acquired from the Breeding Program are quarantined for 10 days, and all trainees undergo initial medical processing (IMP). These procedures include a gastropexy, ear tattoo, and female trainees not intended for breeding also undergo an ovariectomy. After recovery from initial medical processing, MWD trainees then enter a training program to develop them for a working career. Most MWDs are trained to be dual purpose detection and patrol dogs. However, small numbers of single purpose detection and single purpose patrol dogs are also trained as needed. Upon completion of training, MWD trainees are assessed through a qualification process to determine if they are prepared to go to their operational units and kennels. MWD trainees that do not complete training or are unable to pass qualification are either retained as training aids to teach MWD handler students or are discharged from service.

After arrival at an operational kennel, MWDs are paired with a trained MWD handler, and the new MWD team works toward certification. Once certified the MWD team continues maintenance training and is available to support on-base and worldwide requirements. All working animals will inevitably reach a point in their career where they are no longer able to perform the required tasks. When this occurs, medical and behavioral assessments by veterinary personnel are conducted and treatment plans pursued. Should those treatments not be successful in returning the MWD to the required operational capacity, service discharge is pursued (2, 4).

It is unclear how much service discharge is due to cognitive decline in MWDs. Although, the cognitive decline which occurs in humans is well known, research in dogs has found them to have more rapid cognitive decline with age compared to that of humans when tested on discrimination tasks (5). Additionally, increased age in dogs is often accompanied by changes in behavior (6–8). These changes in behavior may be due to the physiological changes which are also occurring with increased age (8–13). Some of these include altered gait, immune responses, respiratory health, and microbiota composition, and these factors may contribute to the decreased performance and working life of MWDs. However, little information exists regarding the age at which these changes are likely to have an effect.

One age related physiological change of special interest in MWDs is the decrease in olfactory acuity. As MWDs rely on their olfaction to detect explosives and drugs and track personnel, the decrement in olfaction associated with age may result in discharge from service. Olfactory abilities have been shown to decrease with age in humans and mice (14–19). Although data supporting age associated olfactory decrement does not exist in dogs, this possible effect should be considered when studying working longevity in MWDs. According to a recent DoD capabilities-based assessment, the required length of service for a MWD is 8 years. Based on currently available information it is unclear if the effects mentioned above are to occur within this 8 year requirement.

Regardless of whether service discharge is due to decreased performance or illness and injury, a decision on the best possible outcome for the MWD is made. Prior to the year 2000, all MWDs either died in service or were euthanized after service discharge. Following policy change, MWDs are now permitted to be adopted by individuals capable of caring for them (3, 4). These individuals frequently include former handlers and community members. Decisions regarding the adoption suitability of MWDs include input from the kennel master, unit commander, and assigned veterinarian. Not all MWDs or MWD trainees are adopted after service discharge. If the MWD or MWD trainee is still a desirable age and has the potential to have a successful working career in a different organization, they can be transferred to other agencies (e.g., fire or law enforcement). If the MWD has a life-threatening condition or a behavioral condition which makes caring for them unsafe for personnel, the MWD is euthanized after service discharge (2).

Any premature service discharge of MWDs represents a loss of assets and loss of investment. Currently, there is a lack of domestic supply of working dogs in the United States, potentially threatening the readiness of agencies utilizing working dogs (20). These factors necessitate the further understanding of the primary causes of MWD service discharge.

Two historical works have reported these findings in MWDs (3, 21). The first report, prior to policy change allowing adoption, reported on causes of MWD death in 1993 to 1996 (21). This report concluded that the majority of deaths and reasons for euthanasia resulted from diseases associated with dogs of advanced age such as neoplasia, degenerative joint disease, and spinal cord disease. The next report of service discharge causes reported on MWDs discharged in years 2000–2004 (3). This work only reported on a select 268 cases, and found behavioral causes, spinal cord disease, and degenerative joint disease to be most prominent.

As the DoD MWD Program has changed since these last reports, updated information regarding the most prevalent causes of MWD service discharge and duration of working life is necessary. The objectives of the presented work are to report causes of MWD service discharge in fiscal years (FY) 2019–2021 and provide information regarding the population demographics of these discharged MWDs. Additionally, this work aims to identify the causes of service discharge and demographic information associated with decreased service life in MWDs.

## 2 Materials and methods

### 2.1 Data acquisition

Initial data retrieval of identification information of MWDs with service discharge dates falling in FY 2019–2021 was conducted using the MWD electronic veterinary medical record (eVMR). Additional information gathered from eVMR included ideal weight range. Additional data retrieval was pursued via MWD disposition review board records and corresponding reports to Congress to identify an exhaustive list of MWDs discharged in FY 2019–2021. Dogs considered MWDs were any procured dogs which passed the pre-purchase examinations and arrived to the 341 TRS or were produced through the DoD MWD Breeding Program. These do not include MWDs who were acquired for or utilized by special operations forces as these dogs have separate management and record keeping. Population information of the MWDs was retrieved from a non-medical MWD database and included sex, breed, source of procurement, goal of procurement, and outcome after discharge. Additionally, dates of procurement and service discharge were also collected for calculation of service life.

### 2.2 Categorization of data

The cause of service discharge was determined using the MWDs’ service discharge request documentation. MWDs with medical reasons of service discharge were categorized and subcategorized by Army Veterinary Corps Officers with extensive MWD experience and relevant specialty training. For medical service discharge causes, veterinary records were used for supporting information. For MWDs with inconclusive diagnoses, the presumptive diagnosis at the time of discharge was used for categorization and subcategorization. Behavior related reasons of service discharge were categorized and subcategorized by an Army Veterinary Corps veterinary behaviorist with extensive MWD experience. A comprehensive list of categories and subcategories with definitions can be found in Supplementary Table 1.

Breeds were categorized as Belgian Malinois, German Shepherd, and Non-typical. Non-typical breeds included any breed making up less than 10% of the MWDs studied. Breeds which were considered non-typical included Labrador Retriever, German Shorthaired Pointer, Dutch Shepherd, Terrier, and other uncommon breeds. The categorized procurement goal of the MWD is the documented work type for which the MWD was originally acquired or bred to complete. This does not necessarily represent the work of the MWD during its service but is rather a reflection of the characteristics selected for during procurement/breeding. Goals included single purpose detection, single purpose patrol, or dual purpose detection and patrol. Detection trained MWDs included both MWDs trained to detect explosives and those trained to detect illicit drugs.

The outcomes after service discharge were categorized as adoption, transfer to another agency, or death. Outcomes of death included euthanasia, natural deaths, and the single MWD who was killed in action in this population. The source of the MWD was categorized as sourced from the DoD MWD Breeding Program or procured from outside sources.

The ideal weight range used for analysis is a range determined by assigned veterinarians during regular examinations and was collected the eVMR. The MWDs’ weight was calculated using the average of the most recent weight range.

Additional data extracted for this population of MWDs included duration of service, service duration compared to the service duration requirement, and operational service duration (for operational dogs). The duration of service was the duration that the MWD was in service and was calculated as the time from when the MWD arrived at the training facility to the date the service discharge was approved. The difference between service life and the 8-year requirement was calculated for each MWD and used for analysis. Additionally, for MWDs who were in the operational subpopulation, operational service life was calculated as the time from when the MWD changed to operational status to the day the service discharge was approved. If the operational status date was unavailable, the day the MWD arrived at its first operational unit was used in its place.

### 2.3 Subpopulations

Three subpopulations within the MWD enterprise were identified and were separated for additional analysis due to differences in management which may impact the causes of service discharge. MWD trainees included any dogs which were bred or purchased but never completed training and were not used as a training aid for MWD handlers. Training aids were MWDs that may or may not have completed training and spent at least half of their service life as a training aid for MWD handlers. Operational MWDs were any MWDs which completed training and went to an operational unit. Operational MWDs may or may not have been training aids but spent less than half their service life as such.

### 2.4 Statistical analysis

#### 2.4.1 Occurrence of category of service discharge

Descriptive statistics were used to report frequencies of sex, breed, goal, source, and subpopulation.

PROC FREQ Chi Square analysis was conducted on frequencies of service discharge categories of all MWDs. Omnibus test significance was determined at 0.05. A follow up pairwise analysis was conducted comparing the five most frequent discharge categories to all other categories. For this additional analysis, a Bonferroni corrected *p*-value was utilized to control type I error. For the 10 most frequent service discharge categories, analysis of frequency of subcategories within those categories was also analyzed via PROC FREQ Chi Square analysis. For this additional analysis, a Bonferroni corrected *p*-value was utilized to control type I error. Chi square analyses were conducted using SAS on Demand.

Logistic regression of occurrence of the five most prevalent service discharge categories was conducted to better identify associations between occurrence of these service discharge categories with population characteristics. Variables of interest were source, goal, breed, outcome, weight, subpopulation, and service life. Odds ratios were utilized for predictive values for significant main effects. This analysis was conducted in R using the GLM method with significance determined at equal to or less than 0.05.

#### 2.4.2 Service life

To visualize the overall service life of the MWDs studied, and to predict survival a Kaplan–Meier survival curve was plotted using the survfit function in R studio. All additional analysis was conducted in SAS on Demand with significance determined at 0.05. To identify variables associated with differing mean service life as well as operational service life, a PROC GLM factorial ANOVA was conducted on service life of MWDs with variables of interest including breed, goal, subpopulation, and outcome of service discharge. Due to limitations in power, the only interaction variable pursued was that of subpopulation and service discharge category. To identify differences in mean service life by subcategory for the five most prevalent categories of service discharge, additional ANOVA tests comparing mean service life by subcategory were conducted. For all ANOVA analysis, Tukey’s honest significant difference was used for pairwise comparisons within significant main effects. Means and standard deviations are reported for these analyses.

Additional descriptive analysis included the total service loss per category. Loss of service life was calculated from the eight year total service requirement for MWDs which was described in a recent DoD capabilities-based assessment. Total loss was calculated as the mean loss in service life multiplied by the number of MWDs discharged for that category. This allowed for the impact of each service discharge category to become observable.

#### 2.4.3 Operational service life analysis

To visualize the operational service life of the MWDs studied and to predict survival by operational service year, a Kaplan–Meier survival curve was plotted using the survfit function in R studio. To identify factors associated with differing means of service, factorial ANOVA in SAS on Demand was conducted to compare operational service life for all operational MWDs. Variables of interest were service discharge category, breed, and goal. As with the other ANOVA analyses, Tukey’s honest significant difference was utilized for pairwise analysis. Significance was determined at 0.05 for all statistical tests. Loss in operational service life could not be calculated as there is no published requirement for operational service life.

## 3 Results

### 3.1 Population description

1,230 MWDs with service discharge dates in FY 2019–2021 were identified. MWDs discharged per fiscal year were 416, 433, and 381, respectively. Of these MWDs, 73.90% (909) were male and 26.10% (321) were female. More of the MWDs were procured from external sources at 84.31% (1,037) compared to those sourced from the MWD Breeding Program at 15.69% (193). The most abundant breed in the population was Belgian Malinois which made up 45.61% (561) of the population, closely followed by German Shepherd which comprised 42.52% (523) of the population. Non-typical breeds contributed the remaining portion. Most [84.19% (1,028)] of the MWDs in the studied population were procured for dual purpose patrol and detection work. Single purpose detection was the goal of 15.56% (190) of the MWDs and single purpose patrol only contributed 0.25% (3) of the population’s goal at procurement.

Adoption was the most common outcome of service discharge for the MWDs studied with 84.23% (1,036) being adopted following discharge. Death accounted for 11.46% (141) of the outcomes with individual causes of death including natural death (43), euthanasia (97) and killed in action (1). 4.31% (53) of the MWDs were transferred to another agency.

Of the 1,230 total MWDs found to have service discharge dates in fiscal years 2019–2021, 931 were operational MWDs (operational), 116 were training aids for MWD handlers (training aids), and 183 were MWD trainees (trainees).

### 3.2 All MWD service discharge categories

**Figure 1:**
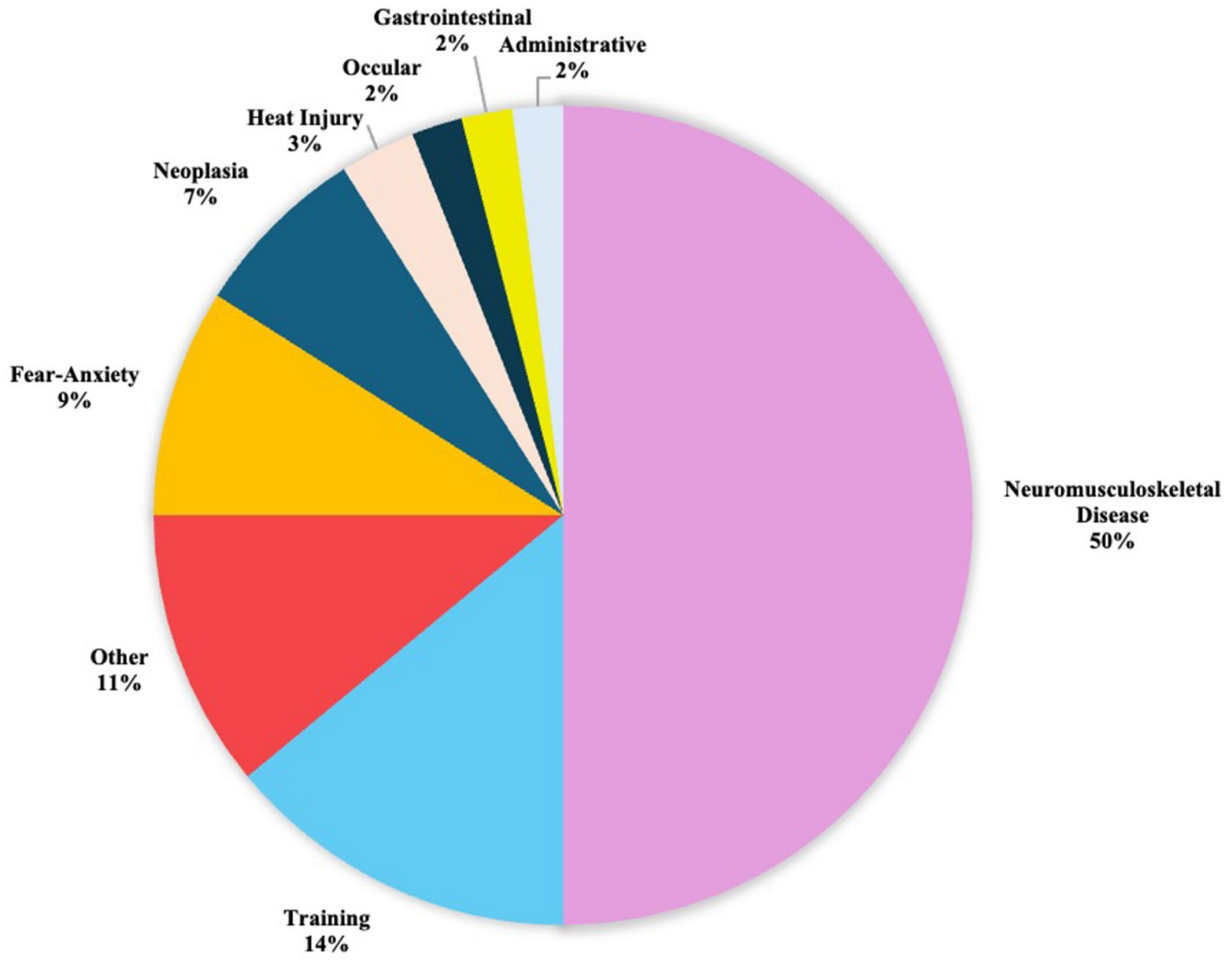
Pie graph depicting the percentages of causes of service discharge in MWDs FY 2019–2021.

Chi square analysis revealed a highly significant difference in proportions by service discharge category (*p* < 0.0001). Follow up analysis used a Bonferroni adjusted alpha of 0.003. The most common service discharge categories were neuromusculoskeletal disease, training, fear-anxiety, neoplasia, and heat injury. The service discharge category with the highest frequency, neuromusculoskeletal disease, was significantly different than all other categories (*p* < 0.003). Frequency of training was significantly different from all other categories (*p* < 0.003). Fear-anxiety frequency was not significantly different from the frequency of neoplasia (*p* = 0.0378) but was significantly different from all other categories (*p* < 0.003). Similarly, neoplasia frequency was significantly different than other categories, excluding fear-anxiety (*p* < 0.003). Heat injury frequency was not significantly different from the frequencies of administrative (*p* = 0.2007), gastrointestinal disease (*p* = 0.2007), and ophthalmologic disease (*p* = 0.2922). However, the frequency of heat injury was significantly different from the other categories (*p* < 0.003). Therefore, these top five categories account for significant proportions of the discharge causes caused when compared to all other categories (Figure 1). Cumulatively, these five most prominent categories account for 83.50% of all the service discharge occurrences in the studied population. Frequencies of each category can be found in Table 1.

**Table 1:** Frequency of occurrence, mean service life, mean service loss, and total service loss in descending order of total service loss for MWDs who were discharged from service during FY 2019 through 2021.

Category	Frequency	Mean service life (years)	Mean service loss (years)	Total lost service (years)
Fear-anxiety^abd^	112	3.40 ± 2.78	−4.6	−514.88
Training^acde^	176	5.20 ± 3.43	−2.8	−492.63
Neuromusculoskeletal disease^bc^	615	7.42 ± 2.27	−0.58	−357.07
Heat injury^ad^	41	3.90 ± 2.51	−4.1	−168.29
Respiratory^ad^	18	3.88 ± 3.07	−4.12	−74.14
Neoplasia^bce^	83	7.16 ± 2.17	−0.94	−69.79
Immune Mediated^ad^	17	4.86 ± 3.12	−3.14	−53.45
Medical behavior^ad^	17	4.96 ± 2.72	−3.04	−51.75
Gastrointestinal disease^ce^	26	6.03 ± 2.78	−1.97	−51.18
Other^abcd^	6	0.99 ± 1.19	−7.01	−42.08
Trauma^a^	13	4.94 ± 2.19	−3.06	−39.84
Ophthalmologic^bce^	32	6.91 ± 2.87	−1.09	−34.89
Cardiovascular^ce^	17	6.74 ± 3.18	−1.26	−21.47
Dermatological^c^	11	6.15 ± 2.14	−1.85	−20.3
Urogenital^ce^	8	6.78 ± 2.02	−1.22	−9.78
Dental^c^	7	6.83 ± 3.18	−1.17	−8.19
Administrative^acd^	26	5.30 ± 1.64	−2.7	−1.97
Infectious^ce^	5	8.25 ± 1.93	0.25	1.24

^a^Indicates significantly different mean service life from neuromusculoskeletal disease (*p* < 0.05). ^b^Indicates significantly different mean service life from training (*p* < 0.05). ^c^Indicates significantly different mean service life from fear-anxiety (*p* < 0.05). ^d^Indicates significantly different mean service life from neoplasia (*p* < 0.05). ^e^Indicates significantly different mean service life from heat injury (*p* < 0.05).

When comparing subcategories within the five most prevalent service discharge categories of the studied population, the categories analyzed were neuromusculoskeletal disease, training, fear-anxiety, and neoplasia (Table 2). The category of heat injury did not have subcategories to analyze, therefore this category was excluded from analysis. An adjusted alpha of 0.01 was used for significance for this follow up analysis. There were differences in frequencies for subcategories within neuromusculoskeletal disease (*p* < 0.0001), with musculoskeletal disease having the highest frequency of 455 (73.98% of category) MWDs. Frequency of subcategory within training significantly differed (*p* < 0.0001), with the most frequent subcategory being task failure. This subcategory caused the discharge of 49.43% (87) of the MWDs in this category. There were significantly more MWDs discharged for fear-anxiety with aggression compared to without (*p* = 0.0045), and this caused the discharge of 71 (63.39% of category) MWDs. Neoplasia did not significantly differ based on the presence of metastasis (*p* = 0.5831).

**Table 2:** Frequency, frequency comparison, mean service life, and service life mean comparison for subcategories of most prevalent service discharge categories for MWDs who were discharged from service during FY 2019 through 2021.

Category	Subcategory	Frequency	Frequency comparison	Service life	Service life comparison
			*p*-value	Mean	*p*-value
Neuromusculoskeletal disease	Combined	112	<0.0001^*^	7.37 ± 2.31	0.2055
	Musculoskeletal	455		7.09 ± 2.19	
	Neurological	48		7.73 ± 2.17	
Training	Stamina Failure_a_	57	<0.0001^*^	6.90 ± 2.68	<0.0001^*^
	Task Failure_b_	87		4.76 ± 3.48	
	Undefined_c_	32		2.38 ± 2.63	
Fear-anxiety	With aggression	71	0.0046^*^	3.58 ± 2.68	0.3323
	Without aggression	41		3.01 ± 3.00	
Neoplasia	With metastasis	44	0.5831	7.21 ± 2.09	0.6957
	Without metastasis	39		7.02 ± 2.32	

^abc^Differing letters indicates significant difference in mean service life. *Indicates statistical difference.

### 3.3 Service discharge category associations

**Figure 2:**
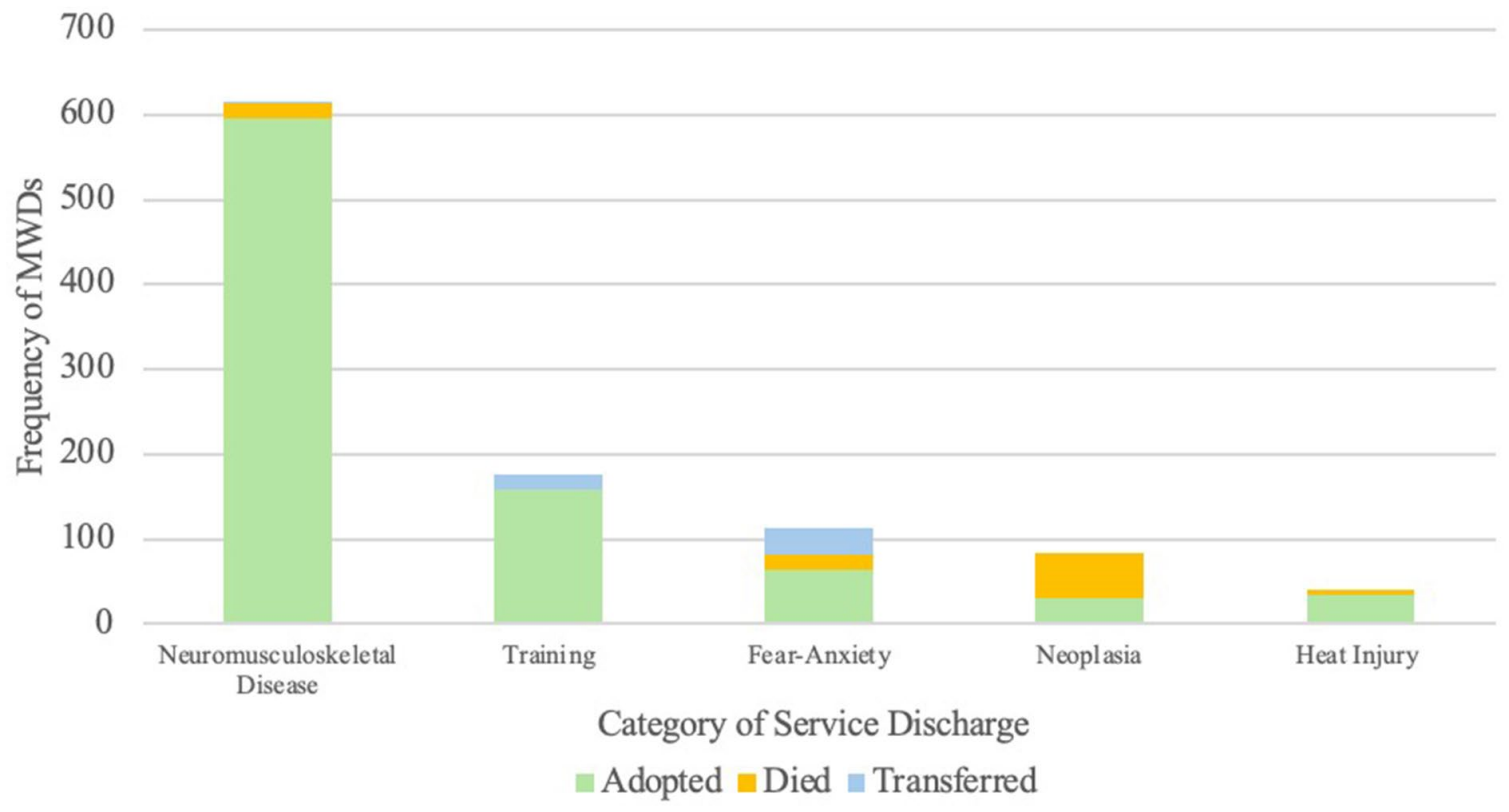
Stacked bar group of frequency of MWD discharges for prevalent service discharge categories differentiated by outcome of service discharge.

When occurrence of each discharge category was analyzed in association with breed, outcome, weight, and service life, several significant associations were discovered. For the most prevalent discharge category, neuromusculoskeletal disease, MWDs who were acquired with a dual purpose goal were significantly more likely to have neuromusculoskeletal disease discharge causes (*p* = 0.0378). MWDs who were acquired for dual purpose patrol and detection had a 91.03% increased likelihood of being discharged for neuromusculoskeletal disease compared to MWDs acquired for single purpose detection goals. Nontypical breeds were significantly less likely to be discharged due to neuromusculoskeletal disease causes (*p* = 0.0092) with a 61.81% decreased likelihood compared to Belgian Malinois. Additionally, the occurrence of neuromusculoskeletal disease service discharge was 94.52% less likely in MWDs with an outcome of death compared to adoption (*p* < 0.0001) (Figure 2). The probability of a neuromusculoskeletal disease service discharge increased by 26.70% with each additional year of service (*p* < 0.0001). This indicates that neuromusculoskeletal disease service discharge causes were likely to occur later in the MWDs’ service life.

When the occurrence of training service discharge causes was analyzed in comparison to other causes, significant effects of goal (*p* < 0.0001), weight (*p* = 0.0327), and outcome (*p* = 0.0122) resulted. MWDs with a goal of dual purpose patrol and detection were 78.05% less likely to be discharged due to training compared to single purpose detection MWDs. When discharged, these MWDs were 280.09% more likely to be transferred to other agencies compared to being adopted. Larger MWDs in this study had a decreased likelihood of service discharge caused by training. The MWDs studied were 6.16% less likely per kilogram increase in ideal weight.

**Figure 3:**
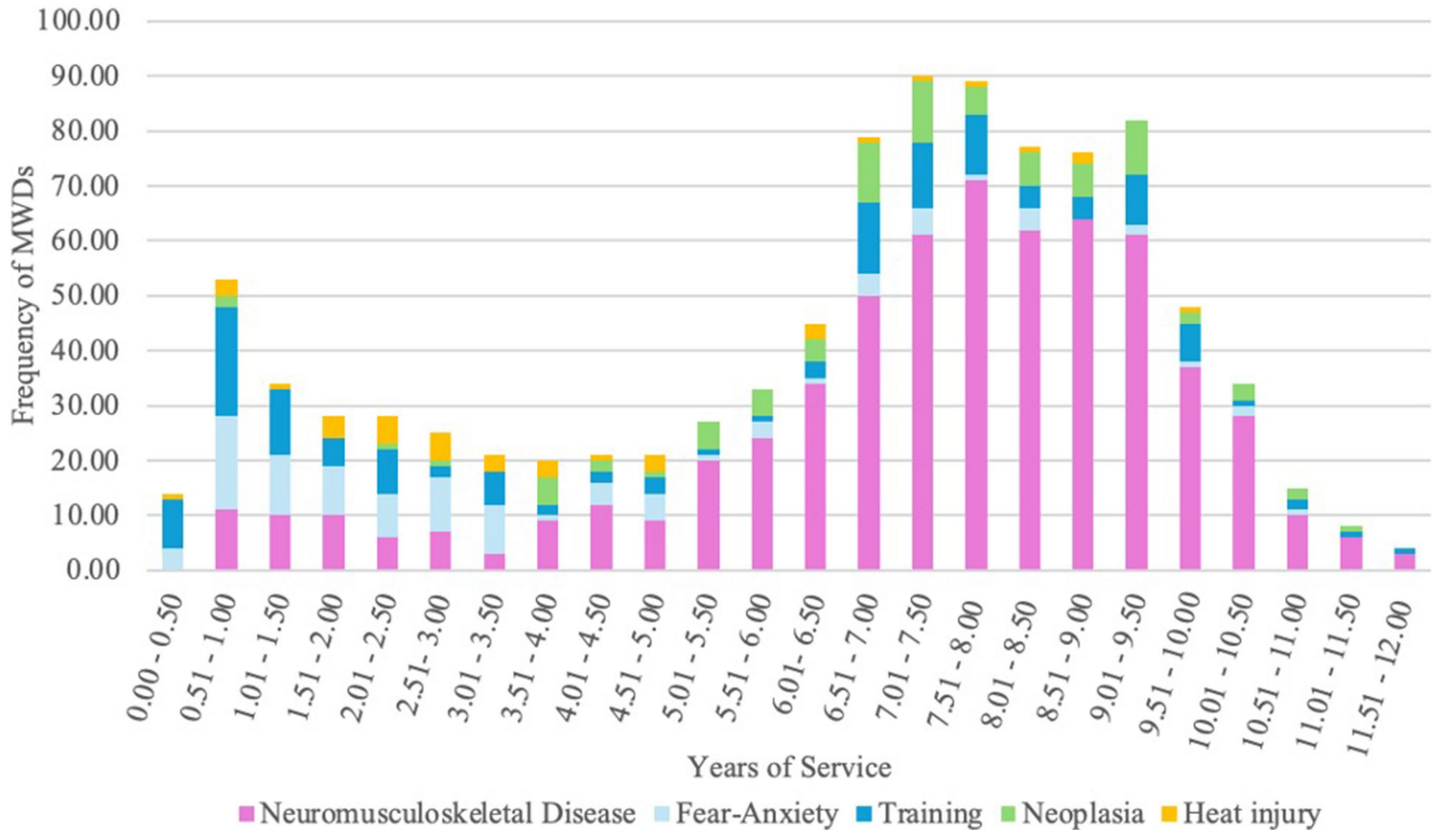
Stacked bar chart of number of MWDs discharged from service per 6 months increment of service life for the most prevalent discharge cause categories.

Similarly, probability of occurrence of fear-anxiety service discharge was significantly associated with main effects of outcome (*p* < 0.0001), breed (*p* = 0.0028), and service life (*p* < 0.0001). There was an increased likelihood of these MWDs being transferred to other agencies (776.58%) or dying (167.62%) compared to being adopted (Figure 2). German Shepherds were 65.24% less likely to be discharged from service for fear-anxiety compared to Belgian Malinois. Additionally, each additional year of service decreased the probability of a fear-anxiety service discharge by 29.01% (Figure 3). This indicates that service discharge for fear-anxiety is likely to occur earlier in service.

Occurrence of service discharge due to neoplasia was significantly more probable in MWDs who died (*p* < 0.0001). The MWDs with neoplasia resulting in service discharge were 2588.39% more likely to die compared to being adopted (Figure 2). Occurrence of neoplasia service discharge increased with length of service life (*p* = 0.0026), resulting in a 27.08% increased likelihood per year (Figure 3). Therefore, neoplasia service discharge was likely to occur later in service.

Heat injury service discharge had occurrences associated significantly with breed (*p* = 0.0245) and service life (*p* = 0.0008). German Shepherds had a 73.98% decreased likelihood of being discharged due to heat injury compared to Belgian Malinois. Each additional year of service decreased the likelihood of heat injury service discharge by 16.31% (Figure 3). This finding indicates that heat injury service discharge is more prevalent in dogs who are discharged earlier in service compared to discharges which occur later in service.

### 3.4 Overall service life

**Figure 4:**
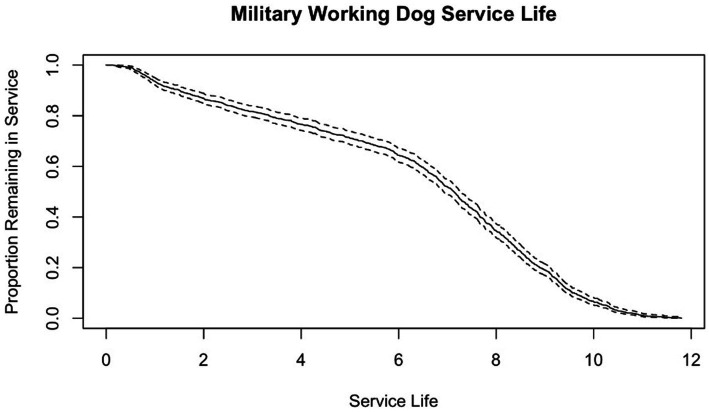
Survival curve of service life of 1,230 MWDs who were discharged from service in FY 2019–2021 with service life measured in years and dotted lines indicating 95% confidence intervals.

**Figure 5:**
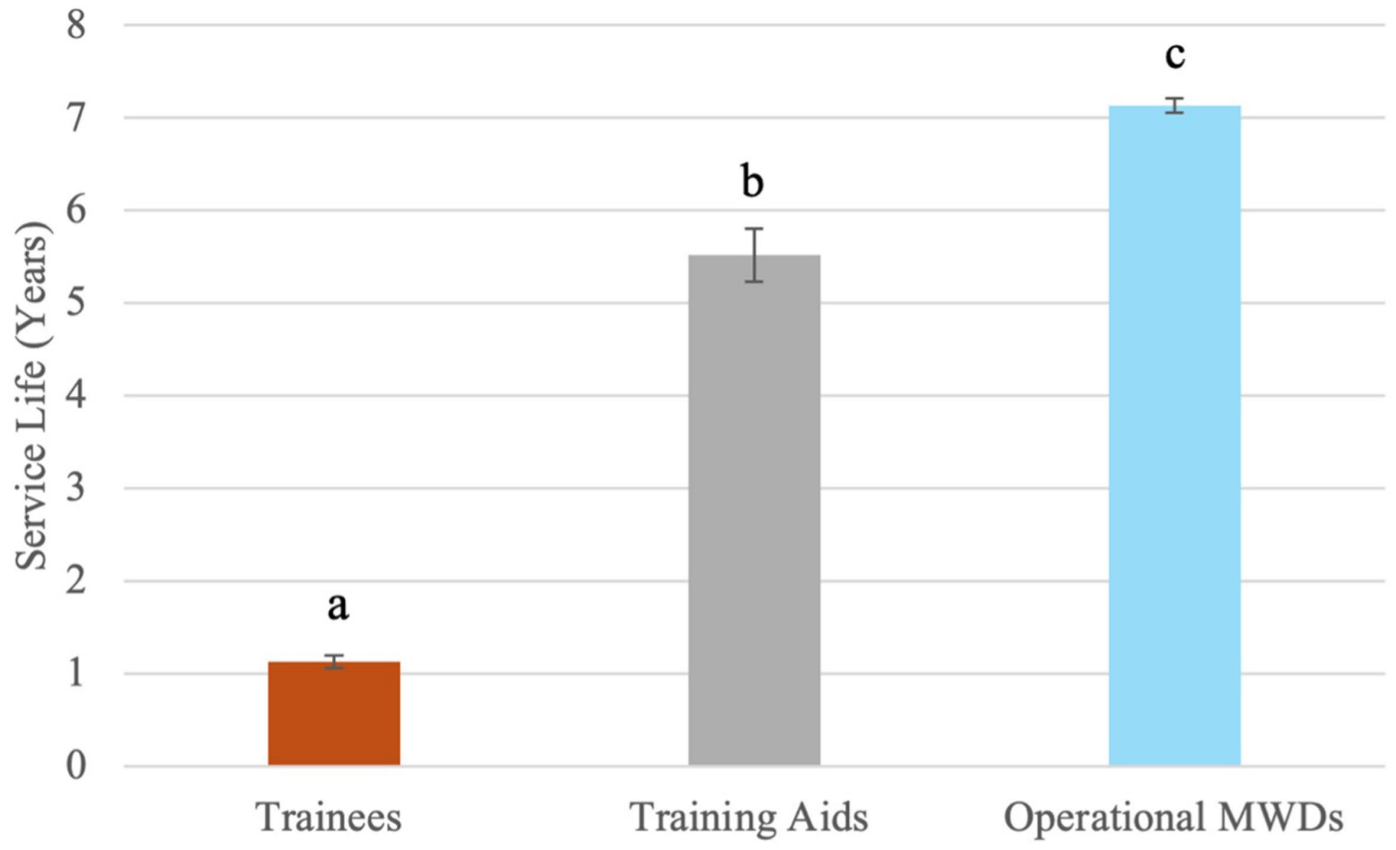
Mean service life for each subpopulation of MWDs with error bars describing standard error. ^abc^Differing letters indicate significant difference in means by subpopulation (*p* < 0.05).

**Figure 6:**
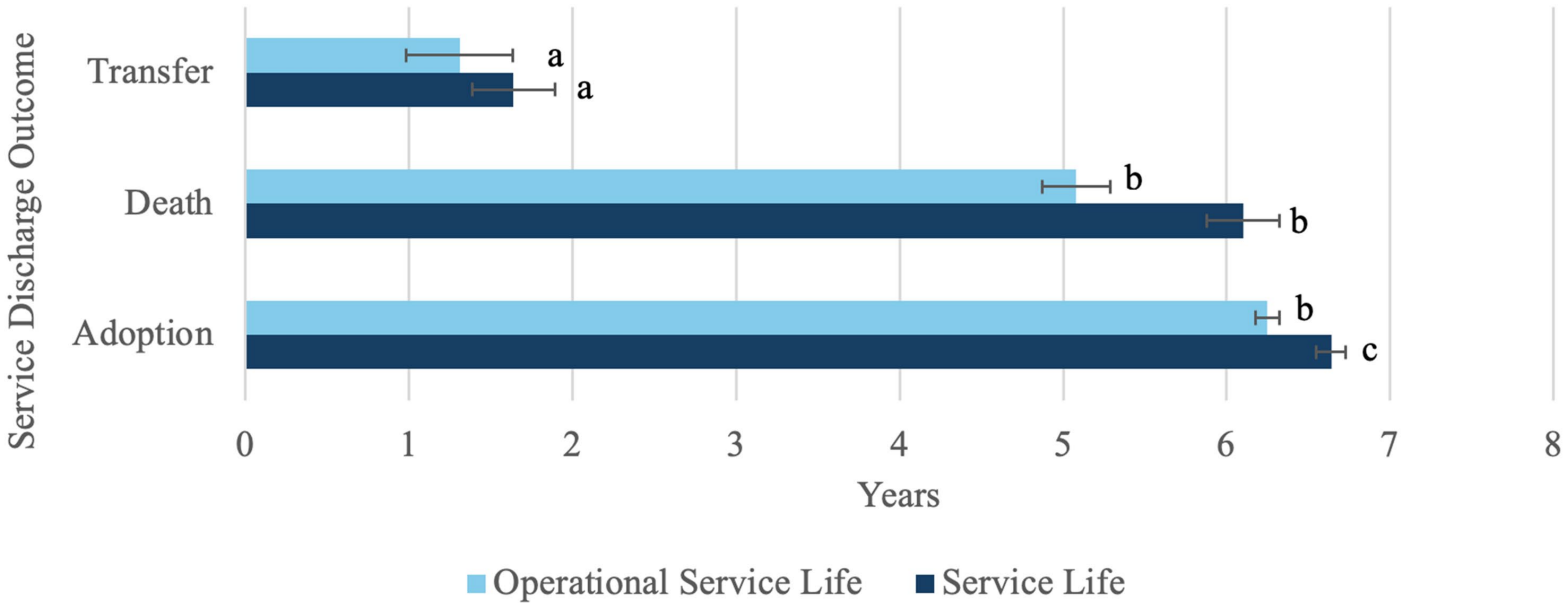
Mean service life and operational service life means by outcome with error bars indicating standard error. ^abc^Differing letters indicate significant difference in means between outcomes within measure (*p* < 0.05).

Of the 1,230 MWDs whose data was collected, 1,150 had the dates necessary in their records to calculate service life. The following results are based on those 1,150 MWDs. Survival curve of MWD service life can be seen in Figure 4. Based on the data, the Kaplan–Meier survival statistic predicts that 34.40% of MWDs will remain in service for 8 years. The overall mean service life was 6.38 ± 2.91 years. When looking at variables associated with changes in mean service life, there were significant associations with main effects of breed (*p* = 0.0252), outcome (*p* = 0.0004), service discharge category (*p* < 0.0001), and subpopulation (p < 0.0001). There was no significant difference by goal (*p* = 0.398). Additionally, there was a significant interaction of service discharge category and subpopulation (*p* = 0.0002) (Figure 5). Pairwise comparisons within breed means indicated German Shepherds had significantly different means compared to Belgian Malinois as well as from nontypical breeds (*p* < 0.05). However, Belgian Malinois and nontypical breeds were similar. Mean service life in years for German Shepherds was 6.64 ± 2.55, for Belgian Malinois was 6.25 ± 3.18, and for non-typical breeds was 5.85 ± 3.06. All pairwise comparisons of outcome were significantly different (*p* < 0.05) (Figure 6). All pairwise comparisons of subpopulations also significantly differed (*p* < 0.0001). Mean service life for all categories can be found in Table 1.

Difference in mean service life by subcategory was analyzed for the most prevalent categories. For neuromusculoskeletal disease, there were no significant differences between musculoskeletal, neurological, or combined musculoskeletal and neurological causes (*p* = 0.2055) (Table 2). For training causes, there were significant differences in service life between the subcategories of stamina failure, task failure, and undefined (*p* < 0.0001). There were no significant differences in mean service life between fear-anxiety causes which presented with aggression versus without (*p* = 0.3323). There was also no difference in mean service life between neoplasia which presented with metastasis and without (*p* = 0.6957). This level of analysis could not be conducted for the heat injury category as there were no subcategories within that category.

The service discharge categories which accounted for the most overall loss of service in FY 2019 through 2021 were fear-anxiety which culminated in a total loss of 171.63 service years per FY (514.88 total), training which resulted in a loss of 164.21 service years per FY (492.63 total), neuromusculoskeletal disease which caused a loss of 119.36 years of service per FY (357.07 total), heat injury which caused 56.10 years of lost service per FY (168.29 total), and respiratory disease which resulted in a loss of 24.71 service years per FY (74.14 total).

### 3.5 Operational service life

**Figure 7:**
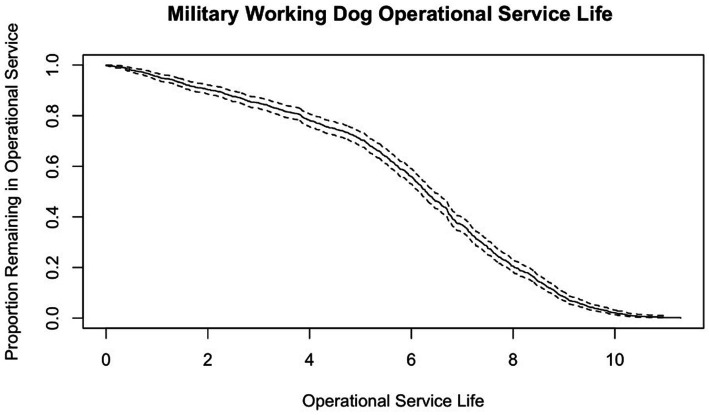
Survival curve of operational service life of 931 MWDs who were discharged from service in FY 2019–2021 with operational service life measured in years and dotted lines indicating 95% confidence intervals.

Of the 1,150 MWDs with calculatable service life, 918 were operational and had calculatable operational service lives. Kaplan–Meier predicted survivals for 10 years of operational service life can be found in Figure 7, and visualization of proportion remaining in service can be found in Figure 7.

When the operational subpopulation was analyzed separately for differences in mean operational service life by variables of interest, breed (*p* = 0.0127), outcome (*p* = 0.0006), goal (*p* = 0.0300), and category (*p* < 0.0001) significantly differed. Belgian Malinois significantly differed from non-typical breeds (*p* < 0.05), but all other pairwise comparisons of breed did not significantly differ. Belgian Malinois had a mean operational service life of 6.27 ± 2.45 years which was more than the mean operational service life of non-typical breeds which was 5.55 ± 2.82 years. Significant pairwise comparisons of outcome included death and transfer compared to adoption (*p* < 0.05). However, death and transfer were not significantly different (Figure 2). Additionally, there was a significant difference between dual purpose and single purpose detection selected MWDs (*p* < 0.05). Single purpose detection MWDs who were operational had a shorter operational service life of 5.65 ± 2.85 years compared to dual purpose MWDs with a service life of 6.18 ± 2.18 years. Multiple pairwise comparisons between categories were significant (*p* < 0.05). Those comparisons significantly different from the most prominent categories are presented in Table 3.

**Table 3:** Mean operational service life for operational MWDs who were discharged from service during FY 2019 through 2021.

Category	Operational frequency	Mean operational service life
Heat Injury^abd^	23	3.17 ± 2.28
Fear-Anxiety^abd^	54	3.85 ± 2.58
Trauma_a_	11	3.97 ± 2.34
Administrative^ad^	25	4.10 ± 1.90
Respiratory	7	4.39 ± 2.78
Medical Behavior	12	5.21 ± 2.23
Immune Mediated	12	5.25 ± 3.25
Gastrointestinal Disease^e^	20	5.54 ± 2.38
Training^ace^	86	5.61 ± 2.83
Cardiovascular^e^	15	5.78 ± 2.53
Urogenital	7	5.79 ± 2.43
Dermatological	9	6.01 ± 1.59
Neoplasia^ce^	75	6.16 ± 2.08
Ophthalmologic^ce^	26	6.44 ± 2.13
Neuromusculoskeletal Disease^be^	540	6.69 ± 1.86
Dental^ce^	5	7.39 ± 1.35
Infectious	3	7.50 ± 3.03
Other	1	8.16

^a^Indicates significantly different mean service life from neuromusculoskeletal disease (*p* < 0.05). ^b^Indicates significantly different mean service life from training (*p* < 0.05). ^c^Indicates significantly different mean service life from fear-anxiety (*p* < 0.05). ^d^Indicates significantly different mean service life from neoplasia (*p* < 0.05). ^e^Indicates significantly different mean service life from heat injury (*p* < 0.05).

## 4 Discussion

The presented work provides results which will support the DoD MWD Program in understanding the prominent causes of discharge and the effects these categories and demographics have on service discharge. However, it is important to note that the description of the presented population is reflective of policies and practices which were occurring as early as 2007 and may not mirror the current MWD population or current MWD enterprise practices.

Adoption was the most prominent outcome following service discharge. However, death still accounted for 11.46% of the MWDs discharged. Outcomes currently cannot be compared to previous literature as all the MWDs studied in 1993 to 1996 were euthanized or died naturally, and the subset of MWDs studied in 2000 to 2004 were all adopted (3, 21). Therefore, this report is novel as it is the first work to report on all discharged MWDs within a period after policy change permitting the adoption of MWDs. Although the majority of the MWDs discharged between 2019 and 2021 were adopted, a more thorough investigation of prominent causes of death and euthanasia should also be conducted and attempts for mitigation should be explored.

In MWDs discharged between 1993 and 1996, the causes which accounted for the highest quantity of MWDs being discharged from service were appendicular degenerative joint disease, neoplasia, and spinal cord disease (21). In the subset of MWDs studied in 2000 to 2004, the causes accounting for more frequent service discharge were behavioral causes, spinal cord disease, and degenerative joint disease (3). In the presented population of MWDs, the most prevalent service discharge causes were categorized as neuromusculoskeletal disease, training, and fear-anxiety. The category of neuromusculoskeletal disease encompasses the degenerative joint and spinal cord disease described in both previous works. The categories of training and fear-anxiety would likely have been identified as behavior in both historical works. Therefore, the findings of the presented work are aligned with previous reporting.

One differentiation to note between the presented and the historical reports is that behavior service discharge was not prevalent in the population studied in 1993 to 1996, and it accounted for only 2% of the reasons for euthanasia (21). Behavior service discharges in the subset studied in 2000 to 2004 were the most frequent in dogs under 5 years old, accounting for 82.30% of the discharges and second most prevalent in dogs 5 years of age and older, accounting for 14.4% of the discharges (3). In the presented work, the behavior related categories of training and fear-anxiety caused more MWDs to be discharged than all other categories except for neuromusculoskeletal disease. For this reason, the researchers of the presented work chose to separate the categories which comprised the historical behavior category in hopes to better identify areas requiring MWD Program focus. The categories selected in the presented work which would have historically been identified as behavioral were training, fear-anxiety, and medical behavior. See Supplementary Table 1 for complete definitions.

Neuromusculoskeletal disease service discharges accounted for 50.00% of the causes studied in the presented population of MWDs. Many of these causes (73.98%) were solely musculoskeletal in nature. Causes which involved both musculoskeletal and neurological processes accounted for 18.21% of the discharges in this category, and solely neurological causes accounted for 7.80%. In the reporting of death and euthanasia in years 1993 to 1996, appendicular degenerative joint disease accounting for 19.20%, and spinal cord disease having accounted for 15.60% of the 927 cases reported (21). In the subset studied in years 2000 to 2004, spinal cord disease alone accounted for 19.59%, degenerative joint disease accounted for 9.39%, and the combination of the two accounted for 8.16% of the 245 MWDs reported (3). Both degenerative joint disease and spinal cord disease would have been categorized as neuromusculoskeletal disease in the analyzed data. Therefore, the presented results are in concurrence with these previous works to the extent that it can be.

Neuromusculoskeletal disease is common in working dogs. Comparing specific disease processes across different working and sporting dog industries can be difficult. However, survey data conducted in the United Kingdom across a wide array of working and performance dog handlers found a high rate of injury at approximately 50% depending on the work/sport (22). A similar survey and examination-based work across multiple sporting and working disciplines found an injury incidence rate of 45.5% (23). Both works mentioned these injuries to primarily be musculoskeletal in nature. In Swedish sporting and utility dogs, 58.7% suffered injuries with muscular, joint, and dermatologic injuries being the most commonly reported (23). These mentioned works included a wide array of activities in dogs likely managed and trained in ways unlikely to directly relate to those used in MWDs. A more appropriate comparison to the presented population is animals used in law enforcement. In 134 law enforcement dogs of New Zealand, the most common cause of retirement was inability to continue with the physical requirements of their work stemming from degenerative musculoskeletal disease (24). This caused the retirement of 45.52% of the dogs studied. It is clear through the presented work as well as others that damage to the neuromusculoskeletal systems account for a significant amount of injuries and loss of working life in working dogs.

Training was the second most abundant category of service discharge in the studied MWDs and caused 14.31% of the service discharges. The MWDs discharged due to training were further subcategorized into task failure (49.43%), stamina failure (32.39%), and undefined (18.18%). Dual purpose MWDs were less likely to be discharged due to training compared to those who were selected for single purpose detection. This may be resultant of the selection process when acquiring MWD trainees. It is possible that the criteria for selecting dual purpose MWDs is more robust compared to that used for single purpose selection. Future work should pursue identification in differences in management and training between dual and single purpose selected MWDs to better identify why their service discharge causes differ.

Interestingly, lighter MWDs were at an increased likelihood of being discharged due to training. Previous work has indicated a relationship between behavior occurrence and weight and height (25). Behaviors that were significantly negatively correlated with weight included excitability, hyperactivity, escaping/roaming, energy, and dog rivalry. Behaviors that were significantly negatively correlated with height included mounting, touch sensitivity, urination, dog-directed fear, non-social fear, owner-directed aggression and more. Interestingly, trainability was positively correlated with weight, indicating that smaller dogs which were studied were less trainable. The referenced work could not identify whether these associations were co-adaptations to size or otherwise caused by unmeasured co-factors. This cited work included a very broad range of breeds which may account for this difference. However, the presented findings of increased training service discharges associated with size and lack of significant association with breed does draw attention to the size of the dog. The association of behaviors and size within working breeds should be evaluated further to discover any possible underlying co-factors such as management differences which may be causing this association. As training service discharge is both abundant and likely to occur early in service, subsequent analysis of this data set will report a more in-depth analysis of these instances and identify associations within the occurrences of the subcategories.

The third most abundant category causing service discharge was fear-anxiety, which caused 9.10% of the discharges reported in this work. Of these, significantly more (63.39%) presented with aggression compared to without aggression (36.61%). In the studied population, German Shepherds were less likely to be discharged from service due to fear-anxiety compared to Belgian Malinois. There is limited work which compares anxiety or other behavioral traits between these two breeds. However, several works have noted the genetic contributions to fear-anxiety related behaviors (26–31), and different breeds have varying amounts of reported aggression (32–34). One work has explored the genotype of a dopamine transporter gene in Belgian Malinois (30). The results found the presence of a specific allele resulted in increased stress behaviors and fewer distraction behaviors. Importantly, this work also reported different handling techniques with dogs of different genotypes as well as different reactions to aversive handling between genotypes. This may provide some explanation to the presented work’s findings. Training techniques, management, and medical conditions have been suggested as contributing factors to anxiety related behaviors in canines (35–44). Although breed and genetic make-up of breeds may be a contributing factor, it is likely other factors may have increased causative impact.

One unexpected finding was the significant association of fear-anxiety service discharge with outcome. These MWDs were more likely to be transferred to other agencies or be euthanized compared to being adopted. Several factors must be considered when interpreting this finding. First of these is the requirement for the MWD to be deemed safe for adoption. MWDs are assessed based on objective data and history, and those with a history and high risk of aggressive behavior are less likely to be considered for adoption to the public. MWDs which are unsafe for the public to adopt or unsafe to be managed by staff for daily maintenance are considered for euthanasia (4). These results regarding outcome may identify a need for increased training of personal in identification of early signs of fear-anxiety and behavior modification to increase the adoptability of more MWDs.

Despite the outcome, the causes and contributing factors associated with the high rate of fear-anxiety discharges should be explored further. Other countries with military working dogs have assessed and observed aggression in their populations (45, 46). One work conducted retrospectively on Israel Defense Force MWDs concluded that bites from MWDs were an occupational hazard that resulted in significant medical burden (46). Regardless of fear-anxiety being present and presenting as aggression in MWDs globally, there may be negative results for the animal. In pet animals, the presence of anxiety resulted in shortened lifespan (47). Previous studies have found impacts of breed, genetic make-up, housing, exercise amount, training practices, and early life experiences all to be contributing to the presence of anxieties in dogs (31, 48–54). To better identify the factors most prevalent to the MWD population studied, additional data collection and analysis is necessary and will be pursued in future reports.

Additional prominent service discharge causes were neoplasia and heat injury. In the reporting from 1993 to 1996, neoplasia accounted for 18.3% of the service discharges and heat stroke accounted for 0.6% (21). In the subset of MWD in 2000 to 2004 heat stroke accounted for 8.2% of the cases reported on Evans et al. (3). However, neoplasia was not a primary cause in this 2000 to 2004 report. It is important to mention again that this referenced work did not include all MWD discharges which occurred within the enterprise, and therefore it is likely that neoplasia related discharges did occur and were just not captured in the subset of cases analyzed. The presented work found 6.75% of the MWDs studied to have been discharged due to neoplasia and 3.33% due to heat injury.

Occurrences of neoplasia service discharge were more likely to result in death in the studied MWDs and were associated with increased duration of service. Although neoplasia still accounted for 6.75% of the presented discharge causes in MWDs, it occurred later during service and was likely a result of increased age. There was also an overall decrease in percentage compared to the amount in 1993 to 1996 which was 18.30% (21). However, this may not be indicative of decreased presence of neoplasia. It is possible that other causes which affect the MWDs earlier in service caused service discharge before the development or discovery of neoplasia. No literature exists currently comparing neoplasia in working animals to non-working. Future research should aim to compare rates of neoplasia in working populations of different disciplines to that of companion animals as well as age of onset to determine if there are additional risks for MWDs.

Interestingly, German Shepherds were at a significantly decreased risk of heat injury discharge compared to the studied Belgian Malinois. A prior work conducted in MWDs which had suffered from heat injury found there to be no association with breed (55). Therefore, future work should explore any genetic or breed related factors which may contribute to the susceptibility of MWDs to heat injury to clarify these conflicting findings. Additionally, the occurrence of heat injury service discharge was likely to occur earlier in the career of the MWD with decreased likelihood of occurrence per additional year of service. This may indicate associations with initial training, the training environment, and more frequent rotation of trainers and handlers earlier in the MWDs’ service life. Regardless of cause or associated factors, mitigation strategies for heat injury have been proposed and are being evaluated. These include cooling vests, water immersion, and physical conditioning (56–60). Although mitigation and preventative strategies are currently being implemented and are in many cases successful, MWDs are still discharged from service due to heat injury. Many MWDs of this cohort were discharged from service due to repeated heat injury events or decreased stamina following heat injury. For some of these MWDs, physical conditioning programs were pursued in attempts to improve heat tolerance but were unsuccessful. Some MWDs included in this work experienced natural death because of heat injury as well. Future work should assess cases of heat injury more closely in MWDs and identify associations, risk factors, and mitigation efforts which may further prevent early service discharge.

As several factors are associated with differing service life, an in-depth analysis of the service life of these MWDs is required. Prominent causes such as training, fear-anxiety, and heat injury which are likely to occur earlier in service may result in increased loss service compared to those which occur later in service. When assessing all categories for average service life, the category with the shortest mean service life was fear-anxiety with a mean service life of 3.40 ± 2.78 years. This category also had the most lost service, accounting for approximately 171.62 years of lost service per FY studied (514.88 years total). Other service discharge categories which had similarly short mean service life were respiratory disease (3.88 ± 3.07) and heat injury (3.90 ± 2.51). The service categories with the longest mean service life were infectious disease (8.25 ± 1.93), neuromusculoskeletal disease (7.42 ± 2.27), and neoplasia (7.16 ± 2.17). Even with their long mean service lives, neuromusculoskeletal disease and neoplasia still accounted for a large portion of the service loss due to the high numbers of MWDs in these categories. This is the first work which has evaluated working life from the perspective of service loss. MWDs differ from other working and sporting dog disciplines as these do not have a universal working life requirement. Future work should identify the cost associated with service life loss to provide an estimate of financial impact associated with each category.

When assessing service life overall, only 34.40% of the MWDs with calculatable service life who were discharged from service during FY 2019 through 2021 stayed in service to the required 8 years. Additionally, the mean service life of the population studied was 6.38 ± 2.91 years which is 1.62 years shorter than the requirement. This is a clear indicator that there were factors which prevented the majority of the MWDs studied from meeting the required service duration.

In published work on police dogs from New Zealand, the mean age of retirement was 7.02 years of age (24). However, in farm dogs of New Zealand, 38% of the dogs studied retired or died in the 10 years or older category (61). These works measured time to retirement in years of age of the dogs that were participating. However, the presented work uses the time the MWDs were in service which is not the same measure as the age of the dogs. MWDs are generally acquired from 12 to 36 months of age, and therefore age of service discharge may be influenced by when the dogs were acquired. Additionally, the DOD MWD program has a vested interest in the duration of MWD working life, rather than at what age the MWDs can work to. However, in comparison to the 7.02 years mean retirement age for the New Zealand police dogs, the presented mean service life of 6.38 years appears to be within reasonable agreement (24). It is possible that the similar work requirements between MWDs and police dogs renders shorter longevity compared to the work of farm dogs. However, additional work is required to distinguish the effects of work on the duration of working life versus effects of breed, management, and training which also vary based on working discipline.

Despite the effects of different work types, the decrease in trained performance seems to be ubiquitous. In a work conducted in humans, performance of job-related training declined with age (62). This was also found in customer service representatives when asked to complete search and retrieval tasks for 3 consecutive days (63). Based on these human findings, it is to be expected that MWDs may also have declining performance as they age. In fact, in work conducted in pets, dogs had more rapid cognitive decline with age compared to humans (5, 8). The presented work was developed to identify contributing factors to decreased service duration that exist outside of age. Age was not assessed in this data set as it is inherently strongly correlated with service duration. Additionally, currently there is no pre-determined age-based work requirement for MWDs, but there is a service duration requirement to which the researchers could compare to. The presented data revealed a difference in service life by breed, service discharge category, goal, and subpopulation. Therefore, the presented work confirms that additional variables exist which contribute to differences service life duration.

When mean service life was compared between German Shepherds, Belgian Malinois, and non-typical breeds, German Shepherds had significantly longer service with a mean of 6.64 ± 2.5 years. Other work on all insured dogs of Sweden assessing risk of morbidity and mortality found that some breeds were more at risk than others (64). This work also found effects of age and sex which were not explored in the presented work. The difference in service life by breed presented may be in part due to an effect of service discharge category. Belgian Malinois had a significantly increased likelihood of being discharged due to fear-anxiety. Fear-anxiety was also likely to cause discharge earlier in service and therefore would result in a decreased service life. Additionally, work comparing Belgian Malinois and German Shepherd deaths from neoplasia noted diagnosis at a younger age, an increased risk of diagnosis, and shorter lifespan in the diagnosed Belgian Malinois group (65). However, analysis of discharge by neoplasia in this population did not note any differences in likelihood associated with breed category. Further work with a more controlled population of working dogs should assess loss of working life between breeds commonly used for detection and patrol purposes. Despite a large sample size in this data collection, the wide variation of MWDs and service discharge causes prevented further statistical analysis of this interaction. In future analyses, controlling for source, age of procurement, selection criteria, working location, and work type could reveal a better trend with breed.

When the significant main effect of subpopulation on service life was explored, the subpopulation of trainees was discharged from service sooner. This was an expected finding as this subpopulation is discharged during initial training and processing. In contrast to trainees, training aids and operational dogs are expected to have similar service life duration. In the studied population, training aids and operational dogs had significantly different mean service life, with training aids having a lower mean service life (5.59 ± 3.08 years) compared to operational dogs (7.13 ± 2.30 years). It is possible that differing management styles or selection standards for these two populations may contribute to this finding. Training aids are frequently MWDs who were deemed unable to function at a full operational capacity, do not have assigned handlers, and have a less consistent schedule compared to operational MWDs. Some work has suggested that housing and human bonding affect the success of working dogs (66, 67). If management practices are a contributor for the studied training aids, the effects may be evident through reduced service life. Further work should explore differences in management practices and their contribution to service life duration.

The results of analysis of operational service life resembled that of the overall service life with some notable differences. Overall service life encompasses all phases of life which includes initial processing and training. Operational service life does not include these initial processes and therefore is a better representation of the time the dog is performing their task. Differences in findings between these two metrics likely indicate differences associated with training and initial intake. There was a significant effect of breed on mean operational service life. When pairwise analysis of the main effect of breed on operational service life was conducted, only Belgian Malinois and non-typical breeds had significantly different means. German Shepherds had a significantly longer overall service life, but they had a similar operational service life compared to the Belgian Malinois. It is possible that these two breeds spent differing amounts of time in initial training but had similar durations of operational life. Differences in trainability, demand, and procurement rates may increase the amount of time that German Shepherds spend at the training center leading to similar operational time. The current work did not assess initial the different portions of the initial intake and training process but should be pursued in the future. This future in-depth analysis should strive to compare time in initial training by breed category to better explain these results.

Procurement goal was a significant main effect for operational service life, but not for overall service life. Single purpose detection selected MWDs had a shorter mean operational service life (5.56 ± 2.85 years) than dual purpose selected MWDs (6.18 ± 2.18 years). This suggests that dual purpose selected MWDs are in operational service longer than single purpose detection selected MWDs. However, their overall service is similar. These findings may be in part due to the lower number of single purpose selected MWDs, the different characteristics that are desired for single purpose MWDs, and the different breeds which may be selected more for single purpose detection. Further work should explore differences in selection criteria and the associated operational service life to better identify selection criteria which promote longevity in service.

The outcome after service discharge was associated with different means of both overall and operational service life. However, pairwise analysis of the outcomes differed for operational service life and overall service life. All outcomes significantly differed by mean overall service life with MWDs who were adopted having the longest overall service life (6.64 ± 2.80 years), MWDs who died having slightly shorter (6.10 ± 2.55 years), and MWDs who were transferred having the shortest (1.64 ± 1.70 years). This was an expected finding as transfer is typically early in the career of the MWD. However, it was unexpected for the mean operational service life of MWDs who were transferred (2.31 ± 1.08 years) and MWDs who died (5.08 ± 2.38 years) to be statistically similar. This is likely due to the difference in population. Most MWDs who are transferred to other agencies are transferred prior to becoming operational, therefore there were only three dogs who were operational and were transferred. With such low frequency this specific comparison was underpowered. Additionally, there is likely an effect of category on the mean service life and operational service life for the group who died. Deaths due to natural causes and age-related conditions such as neoplasia would occur later in service, while behavioral euthanasia is likely to occur earlier.

There is currently no DoD requirement for the duration of operational service. The authors therefore cannot compare the studied operational service life to a requirement. As the time a MWD spends operational is more valuable to the DoD, future work should assess the optimal time required for training and determine an operational service life requirement for MWDs. With this requirement, the variables associated with the greatest loss of operational service can be better identified and assessed more thoroughly.

## 5 Conclusion

One purpose of this work was to identify the most prominent categories causing MWD service discharge. Neuromusculoskeletal disease, training, fear-anxiety, neoplasia and heat injury were identified as the most prevalent service discharge categories and accounted for 83.50% of the total service discharge causes of the studied population. Future work should pursue more in-depth analyses of these major categories to identify more specific causes which contribute to these increased service discharges.

Another objective was to assess and identify possible causes of decreased service duration in MWDs. The mean overall service life did not meet the 8-year service requirement. However, it appears that cause of service discharge, breed, goal, and subpopulation influence the duration of service life. Breed, cause of service discharge, and goal also affect operational service life duration. However, breed and outcome had differing effects on duration of operational service compared to overall service. More work is needed to determine an operational service requirement for U.S. MWDs from which loss can be better assessed. Future work should also pursue identification of methods to increase the longevity of MWDs whether by improved selection, enhanced management, or targeted intervention for prominent service discharge categories.

### 5.1 Study limitations

The limitations of this work include the lack of detailed record review, and the current reported causes of service discharge are based on the stated cause in the discharge request. An in depth medical and training history review was beyond the scope of the resources available. Additionally, the period of study included service discharge which occurred during the COVID-19 pandemic which may have impacted the rate of and decisions regarding service discharge cases which was not captured in the data presented. Future work should compare more recent data to further understand the effects the pandemic had on the service life and service discharge of MWDs. Another variable of interest which could not be explored was that of sex of the dog. This variable was not explored due to the inconsistent timings of alterations of the MWDs. Furthermore, sex data could not be compared to the paired work on service discharge causes which prioritized size of dog over sex. Additional variables which were not included due to incapacity for measurement included handler, deployment, and housing history despite their possible effects on duration of service. Future work should include these variables if they become available.

## Data Availability

The datasets presented in this article are not readily available because of operational security concerns. Requests to access the datasets should be directed to BF, brian.d.farr6.mil@health.mil.
