# Supplementary material for: United States military working dogs from 2019 to 2021: analysis of causes of service discharge and decreased service life
Source: Front Vet Sci. 2025 Oct 9;12:1580628. doi: 10.3389/fvets.2025.1580628 (PMC12548479; doi:10.3389/fvets.2025.1580628)
Supplement: Supplementary file 1 [file Table_1.docx]

Supplementary Material

# Supplementary Table 1. Definitions of service discharge categories for MWD population studied

| **Category** | **Definition** |
| --- | --- |
| Administrative | Discharged for reasons unrelated to disease, injury, behavior, or training (e.g., kennel or unit closing) |
| Cardiovascular Disease | Diagnosed or presumed disease of the cardiovascular system |
| Dental Disease | Diagnosed or presumed disease of the teeth or periodontal tissues |
| Dermatological Disease | Diagnosed or presumed disease of the dermatological system |
| Gastrointestinal Disease | Diagnosed or presumed disease of the digestive tract |
| Fear-Anxiety | Discharged for unmanageable fearful or anxious responses to required operational or environmental stimuli |
| Heat Injury | Diagnosed or presumed disease caused by hyperthermia resulting from external conditions (e.g., not from fever) |
| Immune Mediated Disease | Diagnosed or presumed disease caused by dysregulation of the immune system |
| Infectious Disease | Diagnosed or presumed disease caused by bacteria, viruses, fungi, or parasites |
| Medical Behavior | Diagnosed or presumed psychiatric disease |
| Neoplastic Disease | Diagnosed or presumed disease caused by the abnormal or excessive growth of tissue |
| Neuromusculoskeletal Disease | Diagnosed or presumed disease of the muscular, skeletal, or neurological systems |
| Ophthalmologic Disease | Diagnosed or presumed disease of the ophthalmological system |
| Other | Unknown cause |
| Respiratory Tract Disease | Diagnosed or presumed disease of the respiratory tract |
| Training | Inability or unwillingness to perform a required operational task |
| Trauma | Diagnosed or presumed disease with trauma as the mechanism of injury |
| Urogenital Disease | Diagnosed or presumed disease or the urogenital system |

**
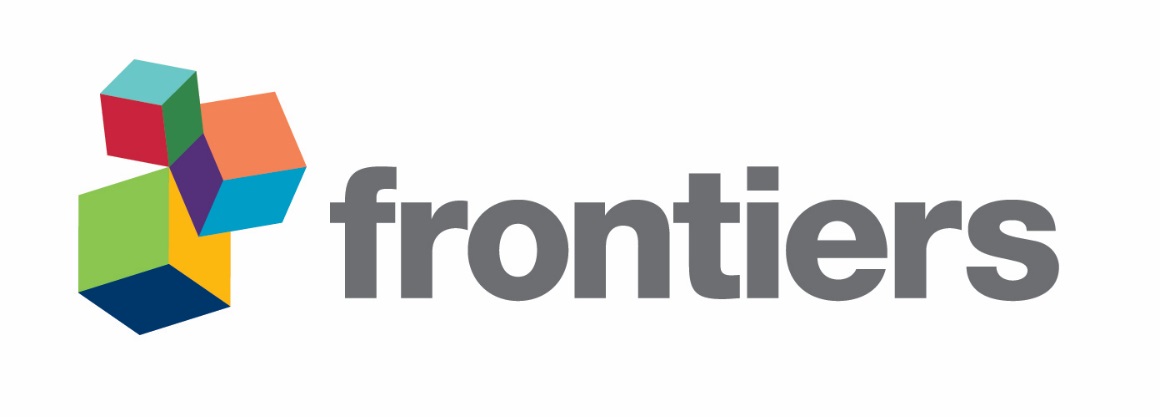
**
